# Relationships of Polychlorinated Biphenyls and Dichlorodiphenyldichloroethylene (*p,p’*-DDE) with Testosterone Levels in Adolescent Males

**DOI:** 10.1289/ehp.1205984

**Published:** 2013-12-20

**Authors:** Lawrence M. Schell, Mia V. Gallo, Glenn D. Deane, Kyrie R. Nelder, Anthony P. DeCaprio, Agnes Jacobs, the Akwesasne Task Force on the Environment.

**Affiliations:** 1 Center for the Elimination of Minority Health Disparities, and; 2 Department of Anthropology, University at Albany, Albany, New York, USA; 3 Department of Epidemiology and Biostatistics, University at Albany, School of Public Health, Rensselaer, New York, USA; 4 Department of Sociology, University at Albany, Albany, New York, USA; 5 Department of Chemistry and Biochemistry, Florida International University, Miami, Florida, USA; 6 Akwesasne Mohawk Nation, Akwesasne, New York, USA

## Abstract

Background: Concern persists over endocrine-disrupting effects of persistent organic pollutants (POPs) on human growth and sexual maturation. Potential effects of toxicant exposures on testosterone levels during puberty are not well characterized.

Objectives: In this study we evaluated the relationship between toxicants [polychlorinated biphenyls (PCBs), dichlorodiphenyldichloroethylene (*p,p´*-DDE), hexachlorobenzene (HCB), and lead] and testosterone levels among 127 Akwesasne Mohawk males 10 to < 17 years of age with documented toxicant exposures.

Methods: Data were collected between February 1996 and January 2000. Fasting blood specimens were collected before breakfast by trained Akwesasne Mohawk staff. Multivariable regression models were used to estimates associations between toxicants and serum testosterone, adjusted for other toxicants, Tanner stage, and potential confounders.

Results: The sum of 16 PCB congeners (Σ16PCBs) that were detected in ≥ 50% of the population was significantly and negatively associated with serum testosterone levels, such that a 10% change in exposure was associated with a 5.6% decrease in testosterone (95% CI: –10.8, –0.5%). Of the 16 congeners, the more persistent ones (Σ8PerPCBs) were related to testosterone, whereas the less persistent ones, possibly reflecting more recent exposure, were not. When PCB congeners were subgrouped, the association was significant for the sum of eight more persistent PCBs (5.7% decrease; 95% CI: –11, –0.4%), and stronger than the sum of six less persistent congeners (3.1% decrease; 95% CI: –7.2, 0.9%). *p,p´*-DDE was positively but not significantly associated with serum testosterone (5.2% increase with a 10% increase in exposure; 95% CI: –0.5, 10.9%). Neither lead nor HCB was significantly associated with testosterone levels.

Conclusions: Exposure to PCBs, particularly the more highly persistent congeners, may negatively influence testosterone levels among adolescent males. The positive relationship between *p,p´*-DDE and testosterone indicates that not all POPs act similarly.

Citation: Schell LM, Gallo MV, Deane GD, Nelder KR, DeCaprio AP, Jacobs A; Akwesasne Task Force on the Environment. 2014. Relationships of polychlorinated biphenyls and dichlorodiphenyldichloroethylene (*p,p´*-DDE) with testosterone levels in adolescent males. Environ Health Perspect 122:304–309; http://dx.doi.org/10.1289/ehp.1205984

## Introduction

During adolescence the reproductive system matures rapidly, driven by changes in the gonadal steroid hormones testosterone and estradiol (Forest et al. 1976). A disturbance in this system during this critical transitional stage may have long-term repercussions because physiologic parameters reached in adolescence are related to endocrine characteristics in adulthood (Root 1973; Roy et al. 2009; Teilmann et al. 2002).

The possibility that exposure to human-made materials could disrupt this important process is of concern. Polychlorinated biphenyls (PCBs) and dichlorodiphenyltrichloroethane (DDT) are lipophilic organochlorines that bioaccumulate in adipose tissue and fat-rich fluids (Carpenter 1998; Wolff and Anderson 1999). PCBs and DDT [or its metabolite dichlorodiphenyldichloroethylene (*p,p´-*DDE)] have been associated with differences in measures of sexual maturation in girls in some studies (Denham et al. 2005; Den Hond et al. 2011; Ouyang et al. 2005; Ozen et al. 2012; Su et al. 2012; Vasiliu et al. 2004; Yang et al. 2005), though not all studies (Gladen et al. 2000; Wolff et al. 2008). Such differences may reflect altered steroid hormone levels and associated functional changes (Bourguignon and Parent 2012). PCBs have been shown to inhibit androgen production *in vitro* (in rats) and *in vivo* (Andric et al. 2000; Kovacevic et al. 1995) possibly mediated by the aryl hydrocarbon receptor (AhR), although some PCB congeners that do not bind to the AhR also have some toxic properties (Fischer et al. 1998; Oskam et al. 2003; Yeowell et al. 1987). In addition to the organic compounds, lead is associated with later sexual maturation in girls (Denham et al. 2005; Kim et al. 1995; Selevan et al. 2003).

Less research has dealt with endocrine disruption of male sexual development by metals or organic compounds. In adults, high levels of lead can interfere with reproduction (Alexander et al. 1996, 1998; Benoff et al. 2003a, 2003b). Some PCB congeners and congener groups were negatively associated with testosterone in a population of Akwesasne Mohawk men with high PCB exposure (Goncharov et al. 2009) and in other adult male populations (Abaci et al. 2009; Dhooge et al. 2006; Pflieger-Bruss et al. 2004). PCBs also have been negatively associated with adult male sperm counts and semen quality (Hauser 2006; Hauser et al. 2003; Mocarelli et al. 2008; Rignell-Hydbom et al. 2004; Toft et al. 2006). These relationships suggest that some persistent organic compounds could affect steroid hormones during adolescent sexual maturation.

Studies of boys’ sexual maturation and its regulating hormones during adolescence have produced suggestive but inconsistent evidence of negative relationships with PCBs and *p,p´-*DDE (Den Hond et al. 2002; Mol et al. 2002; Pflieger-Bruss et al. 2004). The elucidation of effects from exposure to these organics is complicated by the presence of lead that can delay boys’ sexual maturation (Humblet et al. 2011; Korrick et al. 2011; Williams et al. 2010).

To evaluate the role of specific toxicants on aspects of sexual maturation, an approach that models the various exposures suspected of influencing maturation is necessary. The Akwesasne Mohawk Nation is a community with multiple toxicant exposures [PCBs, *p,p´-*DDE, hexachlorobenzene (HCB), lead] and PCB exposures that are substantially greater than those in the U.S. population as a whole (DeCaprio et al. 2005; Gallo et al. 2011; Schell et al. 2003). The aim of the present research is to estimate the relationship of testosterone levels with concurrent levels of PCBs, *p,p´-*DDE, HCB, and lead among male youth with known toxicant exposure while controlling for maturational stage.

## Methods

*Setting*. The study was conducted in partnership with the Akwesasne Mohawk Nation, a sovereign territory that spans the St. Lawrence River, abutting New York State, USA, and Ontario and Quebec, Canada. Industrialization on the St. Lawrence River and some tributaries in the 1950s produced significant contamination. One federal Superfund site and two New York State Superfund sites are proximate to the Akwesasne Nation territory. The U.S. Food and Drug Administration determined that several local species of fish, fowl, and game contained levels of environmental contaminants above levels safe for human consumption (Fitzgerald et al. 1995; Forti et al. 1995; Lacetti 1993; Sloan and Jock 1990). The Akwesasne population’s history of relying on local food sources, which carry the heaviest toxicant burden, suggested that this is a highly exposed population, and this has been confirmed (Schell et al. 2012).

The methods of recruitment, data collection, laboratory analyses, and substitution protocol for toxicant measurements below the limits of detection (LODs) have previously been described in detail (Schell et al. 2003). Members of the Akwesasne Mohawk Nation were trained in data collection techniques by the investigators to collect all anthropometric, interview, and hematological data, but had no prior knowledge of individual’s exposure status. The Institutional Review Board of the University at Albany, State University of New York, approved the study protocols, and interviewers obtained informed assent from each participant and informed consent from the parent/guardian.

*Participants.* Participants were Akwesasne Mohawk youth (10 to < 17 years of age) and their mothers/guardians who lived either on or within 10 miles of the St. Regis Reservation or Reserve between 1996 and 2000. A description of the sampling protocol has been published [see Schell et al. (2003) for details]. The original study sample consisted of 131 males and 140 females and their mothers/guardians. Four males had insufficient serum for testosterone and biomarker assays and were excluded, five were missing lead values, and two were missing triglycerides and cholesterol levels; the final sample was 120 males in the present analysis.

*Blood collection and laboratory analysis*. Fasting blood specimens were collected before breakfast (0700–1030 hours) by trained Akwesasne Mohawk staff. Testosterone level and the time of blood collection were not related (*r* = 0.08, *p* = 0.38). Analyses of the PCBs and organochlorines were conducted by parallel dual-column (splitless injection) gas chromatography with electron capture detection on an Agilent 6890 instrument (Agilent Technologies, Santa Clara, CA) at the University at Albany’s Exposure Assessment Laboratory; the instrument was capable of capturing 83 separate PCB congeners and 18 additional congeners as pairs or triplets, as well as HCB and *p,p´-*DDE (DeCaprio et al. 2000, 2005). Data were expressed on a whole weight basis (i.e., nanograms per gram serum; parts per billion). Blood lead levels were measured using Zeeman-corrected graphite furnace atomic absorption spectrometry (LOD, 1.0 μg/dL; Mercury Monitor Model 100, Pharmacia Corp., Stockholm, Sweden) by Le Centre de Toxicologie du Quebec in Sainte-Foy Quebec, Quebec, Canada. Serum testosterone, estradiol, cholesterol, and triglyceride concentrations were measured by the Clinical Chemistry and Hematology Laboratory, Wadsworth Center for Laboratories and Research, New York State Department of Health (Albany, NY), as described by Schell et al. (2003).

*Testosterone*. Total testosterone was measured in unextracted serum specimens. A specific rabbit antibody affixed to polypropylene tubes (Siemens Diagnostics/Diagnostic Products, Los Angeles, CA) was used in the solid-phase radioimmunoassay procedure. A tracer, ^125^I-labeled testosterone, and a Wallace 1470 Wizard gamma counter (Wallace/PerkinElmer, Waltham, MA) were used to measure radioactivity bound to the tracer. Instrument-based software calculated logit-log transformations, standard curves, and results. The average of duplicate measurements was reported and used. Differences in the duplicate measurements that exceeded 25% (or if the concentration was < 100 ng/dL, then by 25 ng/dL), was the rejection criterion causing re-assay of such specimens. Among samples > 100 ng/dL the variation on duplicate samples was 4.8%, and for samples < 100 ng/dL the variation between duplicates was 7.7%. The functional sensitivity [limit of quantitation (LOQ)] was 10 ng/dL for testosterone. For statistical analyses all results < 10 ng/dL were set to 5 ng/dL, one-half of the LOQ.

*PCBs and organochlorines*. In some participants the levels of some PCBs congeners were below the laboratory LOD or minimum detection limit (MDL). Values below the MDL were imputed for each observation by sampling values from the distribution below the MDL, which was estimated based on the observed data using the method described by Gupta (1952), as recommended by the U.S. Environmental Protection Agency (EPA) for distributions where ≥ 50% of the samples have values above the MDL (U.S. EPA 1998). This method provides a more appropriate representation of the sample variance for values below the MDL than do methods that impute a single value for all samples below the MDL. All values of *p,p´-*DDE and HCB were above the MDL.

Several composite exposure variables were evaluated to allow comparison of our results with those from other studies (following Schell et al. 2003): Σ16PCBs is the sum of all congeners with levels > MDL in ≥ 50% of the study population samples (PCB congeners 52, 70, 74, 84, 87, 95, 99, 101[+90], 105, 110, 118, 149[+123], 138[+163 +164], 153, 180, and 187); Σ8PerPCBs, a persistent PCB group (congeners 74, 99, 105, 118, 138[+163+164], 153, 180, 187); and Σ6NonPerPCBs, a nonpersistent group (congeners 52, 84, 95, 101[+90], 110, and 149[+123]). Brackets indicate “minor” coeluting congeners based on Aroclor concentration (Hansen 1999). We also estimated exposures with estrogenic PCB congeners (Σ7EstrogenicPCBs: congeners 52, 70, 95, 99, 101, 110, and 153), and with PCB-105, an antiestrogenic congener (Cooke et al. 2001; Wolff et al. 1997).

Three composite variables used in a study of adult Akwesasne males were calculated (Goncharov et al. 2009). Using only congeners detected in ≥ 50% of the sample, we calculated mono-*ortho* (Σ4MOPCBs; congeners 118, 105, 74, and 70), di-*ortho* (Σ8DOPCBs; congeners 52, 87, 99, 110, 153, 180, 138[+163+164], and 90[+101]), and tri- and tetra-*ortho* groups (Σ3TTOPCBs; congeners 95, 187, and 84).

*Anthropometric and sociodemographic variables*. Each mother–youth pair completed interviews to report their sociodemographic characteristics, child’s usual diet, whether the child was breastfed, and the mother’s reproductive history (Schell et al. 2003, 2008). Trained staff measured each child’s height and weight following standard anthropometric protocols (Gallo et al. 2005; Lohman et al. 1988). Height and weight *z*-scores were computed using EpiInfo 2000 [Centers for Disease Control and Prevention (CDC) 2001]. Tanner stages (TS) were determined by self-assessment in a private room using drawings employed in a previously validated procedure (Duke et al. 1980; Morris and Udry 1980). Drawings were chosen to provide clearer distinctions between stages and were more acceptable to the community than photographs of the different stages.

*Statistical analysis*. Multivariable linear regression models were used to estimate associations of natural log (ln)–transformed serum testosterone levels with ln-transformed PCB groupings, controlling for other toxicants (*p,p´-*DDE, HCB, lead) and relevant covariates. We included serum HCB concentrations and blood lead levels (both as ln-transformed continuous variables) and whether the child was breastfed as an infant (1 = yes, 0 = no) in all models based on previous research suggesting associations between these factors and serum testosterone (Denham et al. 2005; Goncharov et al. 2009; Schell et al. 2003). In addition, we included the following factors in all models based on correlations with testosterone (*p* < 0.20): TS (categorical as described below), weight-for-age *z*-score and height-for-age *z*-score (both as continuous variables), child cigarette use (1 = yes, 0 = no), child alcohol use (1 = yes, 0 = no), and ln-transformed serum *p,p´-*DDE, cholesterol, and triglycerides (modeled as continuous variables).

The analytical treatment of TS is complicated by its level of measurement (TS is ordinal rather than interval level) and its expected association with age-related covariates. TSs were grouped based on preliminary analysis of variance (ANOVA) models, which indicated that mean testosterone levels were comparable and not significantly different between TS I and II or between TS IV and V, but were significantly different between TS I and II (combined), TS III, and TS IV and V (combined). Therefore, we modeled TS using two indicator variables (for TS I and II, and for TS IV and V) with TS III as the referent category. Standardized measures of weight-for-age and height-for-age *z*-scores reduced age-related confounding.

The functional form of the dose response between ln-transformed PCBs and ln-transformed testosterone was assessed by fitting each exposure as a quadratic polynomial (i.e., by modeling ln-PCBs and a squared term) and using the *p*-value of the squared term as a test for statistically significant departures from linearity (data not shown). All *p*-values were > 0.05; therefore, exposures were modeled as continuous (ln-transformed) variables. Partial-regression plots (i.e., added-variable plots) and partial-residual plots were also examined for evidence of nonlinearity as well as outliers and influence (Fox 2008). Linearity in the dose response was also supported in these plots (data not shown). Nonadditivity (interactions evaluated as product terms and interpreted based on product term *p*-values: We tested all PCBs groups and HCB, *p,p´-*DDE, lead, and breastfeeding history because relatively few studies have examined the concurrent effects of the most common pollutants to which children may be exposed.

To facilitate the interpretation of the results, coefficients were reported in the regression tables, with the coefficients interpreted in the corresponding text. Results are reported as the percent difference in serum testosterone associated with a 10% increase in exposure, which, for ln-transformed exposures, is derived by multiplying the coefficient for the association with ln-transformed testosterone by 10. Statistical analyses were conducted using PASW 19 (IBM 2011). An alpha level of *p* ≤ 0.05 was used to define statistical significance.

## Results

Sample descriptive measures of testosterone, toxicants, covariates, and their patterns of association with TS are shown in Table 1. As expected, mean age consistently increased with increasing TS [*F* = 44.62, df (degrees of freedom) = 122, 4, *p* ≤ 0.001]. Mean testosterone levels also increased with TS (*F* = 59.94, df = 122, 4, *p* ≤ 0.001), with similar mean values in boys classified as TS I or TS II, and as TS IV or TS V. There were 23 boys in TS I and II with testosterone values below the LOQ of 10 ng/dL, and two boys in TS III were below the LOQ. Height increased monotonically with TS (*F* = 32.30, df = 122, 4, *p* ≤ 0.001). Mean weights also differed significantly over TSs (*F* = 7.85, df = 122, 4, *p* ≤ 0.001), yet in contrast with height, this association was not monotonic. Forty-eight percent of the males were breastfed as infants, 9% currently smoked, and 6.7% consumed alcohol.

**Table 1:** Characteristics of adolescent Akwesasne males: mean ± SD for all observations combined (*n* = 120) and by Tanner stage.

Covariates and toxicants^*a*^	All	TS I (*n *= 19)	TS II (*n *= 42)	TS III (*n *= 23)	TS IV (*n *= 32)	TS V (*n *= 11)
Age (years)	13.17 ± 1.95	11.32 ± 1.09	11.93 ± 1.43	13.31 ± 1.51	14.62 ± 1.09	16.04 ± 0.81
Testosterone (ng/dL)^*b*^	252.18 ± 279.60	26.41 ± 35.31	48.71 ± 90.71	264.70 ± 252.28	509.88 ± 203.42	555.70 ± 299.24
Height (cm)	157.97 ± 13.39	148.83 ± 8.27	149.79 ± 10.61	156.53 ± 10.45	167.46 ± 9.10	177.56 ± 6.35
Height-for-age *z*-score	0.12 ± 1.20	0.50 ± 0.93	–0.01 ± 1.29	–0.03 ± 0.91	–0.04 ± 1.41	0.83 ± 0.82
Weight (kg)	62.59 ± 19.04	61.56 ± 20.95	53.37 ± 18.75	62.22 ± 14.54	68.16 ± 16.68	82.39 ± 13.51
Weight-for-age *z*-score	1.48 ± 1.63	2.73 ± 2.17	1.26 ± 1.71	1.37 ± 1.13	1.13 ± 1.41	1.59 ± 1.08
Triglycerides (mg/dL)	84.65 ± 45.82	103.29 ± 56.19	100.71 ± 51.79	74.17 ± 42.23	68.38 ± 27.56	68.10 ± 29.49
Cholesterol (mg/dL)	158.68 ± 32.80	163.12 ± 35.69	171.18 ± 34.88	161.30 ± 32.75	146.66 ± 25.61	136.00 ± 16.63
Σ16PCBs	0.77 ± 0.41	0.77 ± 0.37	0.88 ± 0.56	0.60 ± 0.21	0.75 ± 0.35	0.74 ± 0.17
Σ8PerPCBs	0.47 ± 0.30	0.41 ± 0.15	0.55 ± 0.43	0.38 ± 0.15	0.46 ± 0.25	0.46 ± 0.16
Σ6NonPerPCBs	0.24 ± 0.16	0.29 ± 0.19	0.26 ± 0.21	0.17 ± 0.07	0.23 ± 0.12	0.22 ± 0.06
Σ7EstrogenicPCBs	0.38 ± 0.22	0.40 ± 0.23	0.44 ± 0.29	0.29 ± 0.11	0.37 ± 0.17	0.35 ± 0.08
Antiestrogenic PCB	0.02 ± 0.02	0.02 ± 0.01	0.03 ± 0.02	0.02 ± 0.02	0.03 ± 0.01	0.02 ± 0.01
Σ4MOPCBs	0.15 ± 0.09	0.16 ± 0.07	0.17 ± 0.13	0.13 ± 0.06	0.15 ± 0.07	0.14 ± 0.04
Σ8DOPCBs	0.51 ± 0.29	0.51 ± 0.26	0.60 ± 0.39	0.40 ± 0.16	0.50 ± 0.25	0.51 ± 0.15
Σ3TTOPCBs	0.08 ± 0.04	0.08 ± 0.04	0.09 ± 0.05	0.06 ± 0.02	0.07 ± 0.03	0.08 ± 0.01
*p,p’*-DDE	0.45 ± 0.35	0.31 ± 0.11	0.48 ± 0.50	0.40 ± 0.19	0.51 ± 0.35	0.52 ± 0.25
HCB	0.04 ± 0.02	0.03 ± 0.02	0.04 ± 0.03	0.04 ± 0.02	0.05 ± 0.03	0.03 ± 0.01
Lead (μg/dL)	1.59 ± 0.97	1.48 ± 0.72	1.67 ± 0.94	1.25 ± 0.93	1.79 ± 1.03	1.70 ± 1.23
Σ16PCB: congeners with ≥ 50% detection rate, sum of PCBs 52, 70, 74, 84, 87, 95, 99, 101[+90], 105, 110, 118, 138[+163+164], 149[+123], 153, 180, 187; Σ8PerPCBs: sum of PCBs 74, 99, 105, 118, 138[+163+164], 153, 180, 187; Σ6NonPerPCBs: sum of PCBs 52, 84, 95, 101[+90], 110, 149[+123]; Σ7EstrogenicPCBs: sum of PCBs 52, 70, 95, 99, 101[+90], 110, 153 (Cooke et al. 2001); antiestrogenic PCB: PCB-105 (Cooke et al. 2001); Σ4MOPCBs: sum of PCBs 70, 74, 105, 118 (Goncharov et al. 2009); Σ8DOPCBs: sum of PCBs 52, 87, 99, 110, 138[+163+164], 153, 101[+90] (Goncharov et al. 2009); Σ3TTOPCBs: sum of PCBs 84, 95, 187 (Goncharov et al. 2009). Brackets indicate “minor” coeluting congener based on Aroclor concentration (Hansen 1999). ^***a***^Values < MDL were imputed from the estimated distribution < MDL (ppb unless otherwise indicated). ^***b***^The functional sensitivity for testosterone was 10 ng/dL; for statistical purposes, results below the LOQ was set at 5 ng/dL.						

Mean serum PCB, *p,p´-*DDE, HCB, and lead concentrations were similar to values previously reported for the combined sample of Akwesasne male and female adolescents (Schell et al. 2003). The geometric mean concentration of Σ8PerPCBs (0.41 ppb) measured in 1996–2000 in our study population of 10- to < 17-year-old Akwesasne males exceeded the 95th centile (0.40 ppb) of the same congeners (74, 99, 105, 118, 138 [163 + 164], 153, 180, 187) reported by the CDC for 12- to 19-year-old males and females based on NHANES (National Health and Nutrition Examination Study) data collected during 1999–2004 (CDC 2009). Geometric mean *p,p´-*DDE and HCB concentrations (0.39 ppb and 0.04 ppb, respectively) were lower than corresponding values reported by the CDC (1.69 ppb and 0.07 ppb, respectively).

Associations among toxicants and congeners were also examined (see Supplemental Material, Methods, Table S1). As expected, the different PCB congener groups were highly intercorrelated, reflecting the inclusion of many of the same congeners (range of *r* = 0.53–0.94). The levels of Σ16PCBs, Σ8PerPCBs, and Σ7EstrogenicPCBs were correlated with the level of *p,p´-*DDE (*r* = 0.43, 0.56, and 0.34 respectively, *p* < 0.01). HCB was correlated with *p,p´-*DDE (*r* = 0.41, *p* ≤ 0.01) and marginally with Σ8PerPCBs (*r* = 0.20, *p* ≤ 0.05). Nonpersistent PCB groupings and the antiestrogenic PCBs were not significantly correlated with *p,p´-*DDE. Lead was not correlated with any of the PCB variables or with *p,p´-*DDE or HCB.

*Testosterone and toxicant levels*. Multivariable regression indicated a significant negative association between Σ16PCBs and serum testosterone, such that a 10% increase in Σ16PCBs was associated with a 5.6% decrease in testosterone (95% CI: –10.8, –0.5%) (for complete model results, including associations with model covariates, see Supplemental Material, Table S2). There was a positive though nonsignificant association between *p,p´-*DDE and testosterone based on the same model, such that a 10% increase in *p,p´-*DDE was associated with a 5.2% increase in testosterone (95% CI: –0.5, 10.9%, *p* = 0.07).

Other indices of PCB exposure (Σ8PerPCBs and congener groups used in the analysis of adult Akwesasne males) were tested using the same multivariable model (Table 2). Testosterone was negatively associated with Σ8PerPCBs, Σ4MOPCBs, and Σ7EstrogenicPCBs. For every 10% increase in these PCB groups, mean testosterone levels were 5.7% (95% CI: –11.0, –0.4%), 6.2% (95% CI: –11.2, –1.2%), and 4.7% (95% CI: –9.2, –0.1%) lower, respectively. Associations with Σ8DOPCBs and Σ3TTOPCBs were also negative, but not significant (–4.6%; 95% CI: –9.3, 0.1%, *p* = 0.06 and –4.8% 95% CI: –10.3, 0.7%, *p* = 0.09, respectively). The nonpersistent PCBs and the antiestrogenic PCB-105 also were negatively but not significantly associated with testosterone.

**Table 2:** Predictors of testosterone levels in adolescent males: results of the multivariable regression analysis with PCB groupings (*n* = 120).

Toxicant (ppb)^*a*^	β (95% CI)	*p*-Value	Percent change^*b*^
Σ16PCBs	–0.56 (–1.08, –0.05)	0.03	5.6
Σ8PerPCBs	–0.57 (–1.10, –0.04)	0.03	5.7
Σ6NonPerPCBs	–0.31 (–0.72, 0.09)	0.13	3.1
Σ7EstrogenicPCBs	–0.47 (–0.92, –0.01)	0.05	4.7
Antiestrogenic PCB	–0.15 (–0.59, 0.29)	0.50	1.5
Σ4MOPCBs	–0.62 (–1.12, –0.12)	0.02	6.2
Σ8DOPCBs	–0.46 (–0.93, 0.01)	0.06	4.6
Σ3TTOPCBs	–0.48 (–1.03, 0.07)	0.09	4.8
Each model controlled for Tanner stages (TS) I and II vs. III, TS IV and V vs. III, weight-for-age *z*-score, height-for-age *z*-score, breastfed as an infant (yes/no), child’s alcohol use (yes/no), child’s cigarette use (yes/no), triglycerides (mg/dL), cholesterol (mg/dL), lead (μ/dL), *p,p’*-DDE (ppb), and HCB (ppb). Testosterone, cholesterol, triglycerides, lead, *p,p’*-DDE, HCB and PCB variables are ln-transformed. Σ16PCB: congeners with ≥ 50% detection rate, sum of PCBs 52, 70, 74, 84, 87, 95, 99, 101[+90], 105, 110, 118, 138[+163+164], 149[+123], 153, 180, 187; Σ8PerPCBs: sum of PCBs 74, 99, 105, 118, 138[+163+164], 153, 180, 187; Σ6NonPerPCBs: sum of PCBs 52, 84, 95, 101[+90], 110, 149[+123]; Σ7EstrogenicPCBs: sum of PCBs 52, 70, 95, 99, 101[+90], 110, 153 (Cooke et al. 2001); antiestrogenic PCB: PCB-105 (Cooke et al. 2001); Σ4MOPCBs: sum of PCBs 70, 74, 105, 118 (Goncharov et al. 2009); Σ8DOPCBs: sum of PCBs 52, 87, 99, 110, 138[+163+164], 153, 101[+90] (Goncharov et al. 2009); Σ3TTOPCBs: sum of PCBs 84, 95, 187 (Goncharov et al. 2009). Brackets indicate “minor” coeluting congener based on Aroclor concentration (Hansen, 1999). ^***a***^Values < MDL were imputed from the estimated distribution < MDL. ^***b***^Percent change associated with a 10% increase in exposure.			

There was no clear evidence of nonadditivity between PCBs and HCBs, *p´p-*DDE, or lead on associations with testosterone (all interaction *p-*values > 0.30). Associations between PCBs and testosterone were stronger in boys who were not breastfed compared with boys who were breastfed, though interactions also were not significant. For example, a 10% increase in Σ16PCBs was associated with a 9.7% decrease in testosterone (95% CI: –19.6, 0.2%) among boys who were not breastfed, compared with a 4.5% decrease (95% CI: –10.7, 1.6%) among boys who were breastfed (the interaction of breast feeding by Σ16PCBs was not significant; *p* = 0.80).

## Discussion

Among male Mohawk youth, testosterone was negatively associated with several groupings of PCB congeners, and positively associated with *p,p´-*DDE. Because this is an observational, cross-sectional study, causality cannot be inferred, and, given the sample size, these observations should be tested in other samples.

The results are strengthened by certain features of the study. First, although focusing on PCBs, we were able to consider several other common toxicants, including a heavy metal (lead), and two pesticides (HCB and *p,p´-*DDE). Also, this study employed congener-specific laboratory analysis that allowed the calculation of PCB levels in subgroups of congeners. This enabled comparisons of associations between PCB subgroups that might differ in structure and/or persistence with testosterone. The more persistent PCBs were clearly associated negatively with testosterone, whereas nonpersistent PCBs were not. The lack of relationships with the nonpersistent congeners suggests that current exposure is not influential or is not great enough to have an observable effect.

Congener-specific laboratory analyses also allowed us to replicate the same structure-based PCB groups as Goncharov et al. (2009) used in their study of Akwesasne men; our results in youth were similar in direction and statistical significance, suggesting that the adult profile may result from a developmental trajectory evident in adolescence. Furthermore, we were able to test several PCB congener groups used by other investigators to evaluate replicability across studies. It is important to recognize that the congener composition of the PCB composite variables overlap (see footnote of Tables 1 and 2).

An additional strength of this study is the use of TSs as an independent variable in lieu of age or height, which vary widely with markers of maturation during adolescence, and thus are a poor proxy for maturation in statistical models (Tanner 1962). Because timing of maturation itself could be affected by these toxicants, it is important to remove the effect of timing on testosterone levels. By statistically adjusting for maturation as indexed by TS, it is possible to estimate the effects of toxicants on testosterone independent of the effects on timing of sexual maturation.

A weakness, however, is the self-assessment of TS. Some researchers have found significant correlations between self-assessed TSs and hormonal development (Shirtcliff et al. 2009), whereas others have found that male adolescents both under- and overestimate their TS (Desmangles et al. 2006; Taylor et al. 2001; Williams et al. 1988). However, to the best of our knowledge, there is no evidence that over- or underestimation varies with toxicant exposure. Without such evidence, we believe that the error is most likely unbiased, although bias cannot be ruled out.

Negative associations between PCBs and testosterone were weaker for less persistent congeners than for more persistent congeners, which suggests that earlier exposures may be more relevant to the associations. However, it is not possible to assess the temporal relation between exposures and the outcome, given the cross-sectional study design. Finally, because the Akwesasne Mohawk Nation is not federally censused, it is not possible to know what proportion of the community between 10 and < 17 years of age is sampled.

The clinical relevance of the differences in testosterone associated with *p,p´-*DDE and PCB exposures in the study population is not known, because reference values for serum testosterone concentrations are not available (Kronenberg and Williams 2008). Nevertheless, associations between environmental exposures and testosterone levels in the study population are a concern, given that adolescence is a critical period for the establishment of adult hormone homeostasis (Root 1973). Similar negative associations estimated between PCBs and testosterone in adult Mohawk men from the same community suggest the possibility of early-life effects that may persist into adulthood (Goncharov et al. 2009).

An interesting finding is that the associations of PCBs and *p,p´-*DDE with testosterone were stronger among boys who had not been breastfed. It is difficult to attribute the weaker effect among the breastfed youth to a moderating effect of breastfeeding because breastfeeding delivers additional exposure to PCBs and other lipophilic compounds (Greizerstein et al. 1999). In the Akwesasne sample here, the level of PCBs was significantly higher among those who had been breastfed compared with those not breastfed (Gallo et al. 2011; Schell et al. 2003). Toxicants delivered through lactation may not influence some end points if the period of developmental sensitivity of an end point has passed. In such circumstances, lactation essentially delivers an additional toxicant burden randomly with regard to the dependent variable. This may obfuscate the relationship rather than mitigate it. This interpretation is consistent with results regarding thyroid hormones (Schell et al. 2009), but is limited by the small samples of breastfed and not breastfed boys.

Several studies have examined such relationships among boys within a narrow age range. Studies of Flemish adolescents, 14–15 years of age, from areas differing in exposure characteristics examined hormone and toxicant levels measured concurrently. These studies have produced evidence of both stimulated and decreased testosterone in relation to marker PCBs and pollution, but no associations with *p,p´-*DDE (Croes et al. 2009; Den Hond et al. 2002; Dhooge et al. 2011). Finally, a study of neonatal hexachlorobiphenyl exposure in rats found decreased adult serum testosterone levels (Xiao et al. 2011).

Three studies have evaluated relationships between pubertal development and prenatal exposure to PCBs, PCDFs (polychlorinated dibenzofurans), or *p,p´-*DDE. In a longitudinal study of 304 singleton males born in the early 1960s and followed through adolescence (Gladen et al. 2004), *p,p´-*DDE levels in cord blood were not associated with testosterone (*p-*values for all tests > 0.10). There was some evidence of reduced pubertal testosterone in Yucheng boys (*n* = 21) who had experienced an acute prenatal exposure to a mixture of PCBs and PCDFs when they were compared with matched controls (Hsu et al. 2005). PCB levels measured in cord blood (PCB-138[+163+164], 153, and 180 combined) were not associated with serum testosterone in Faroese boys at 13–14 years of age (Mol et al. 2002). In our analysis, serum PCB and *p,p´-*DDE concentrations were not highly correlated, and mutually adjusted associations with testosterone were in opposite directions.

Two studies of adult men reported significant negative associations between some PCBs (mono-, tri-, and di-*ortho* substituted PCBs) and testosterone (Goncharov et al. 2009; Richthoff et al. 2003). Great Lakes sport fish consumers’ PCB levels were negatively associated with SHBG (sex hormone binding globulin)–bound testosterone, though not with total or free testosterone (Persky et al. 2001; Turyk et al. 2006). Other studies of human populations have produced evidence suggesting relationships of organochlorines to measures of reproductive hormones or function (Ferguson et al. 2012; Richthoff et al. 2003; Rignell-Hydbom et al. 2004), or no evidence of a relationship in adult men (Hagmar et al. 2001). Of the studies of adult men with PCB exposures (Ferguson et al. 2012; Goncharov et al. 2009; Richthoff et al. 2003; Rignell-Hydbom et al. 2004), the study of Akwesasne men with high exposure has provided the strongest evidence for a relationship of testosterone with PCBs (Goncharov et al. 2009). Serum PCB levels measured in our study population of Akwesasne boys in 1996–2000 were higher than serum levels measured in a representative sample of U.S. adolescents (age 12–19 years) in 1999–2004, but similar to levels measured among Akwesasne men during the same time period (Goncharov et al. 2009). Differences in exposures among populations could explain differences in associations among studies (Goncharov et al. 2009).

## Conclusion

In this study we found significant associations between testosterone and Σ16PCBs, with stronger associations estimated for more persistent congeners, and *p,p´-*DDE in a population of adolescent Native American males with relatively high PCB exposures. These results are consistent with experimental studies in animals that found endocrine-disrupting chemicals, such as PCBs and *p,p´-*DDE, modified serum testosterone levels (Ahmad et al. 2003; Andric et al. 2000; Kovacevic et al. 1995; Xiao et al. 2011), and with studies of humans that found associations consistent with endocrine modification due to those exposures (Croes et al. 2009; Dhooge et al. 2011; Goncharov et al. 2009; Persky et al. 2001; Richthoff et al. 2003; Turyk et al. 2006).

## Supplemental Material

(102 KB) PDF
