# Supplementary material for: Relationships of Polychlorinated Biphenyls and Dichlorodiphenyldichloroethylene (p,p’-DDE) with Testosterone Levels in Adolescent Males
Source: Environ Health Perspect. 2013 Dec 20;122(3):304–9. doi: 10.1289/ehp.1205984 (PMC3948020; doi:10.1289/ehp.1205984)
Supplement: (102 KB) PDF [file ehp.1205984.s001.pdf]

Environmental Health Perspectives doi:10.1289/ehp.1205984

Supplemental Material
Relationships of Polychlorinated Biphenyls and Dichlorodiphenyldichloroethylene (*p,p'*-DDE) with Testosterone Levels in Adolescent Males

Lawrence M. Schell, Mia V. Gallo, Glenn D. Deane, Kyrie R. Nelder, Anthony P. DeCaprio, Agnes Jacobs, and the Akwesasne Task Force on the Environment

Table of Contents

Methods: Analyses of the PCBs and organochlorines ……………………………………........p. 2

References……………………………………………………………………………………p. 3

Supplemental Material, Table S1………………………..……………………………….....p. 4

Supplemental Material, Table S2. ………………………………………………………......p. 5

Methods

Analyses of the PCBs and organochlorines

The analytical reproducibility of the method for PCB analysis is fully described and discussed in the DeCaprio et al. (2000) and (2005) papers. In general, the %RSD (relative standard deviation) for all congeners was < 20%, with most < 15%, which is acceptable for ultra-trace determinations.

Correlations of the different toxicants were determined by bivariate analysis (Supplemental Table S1). As expected, the different PCB congener groups were highly intercorrelated reflecting the inclusion of many of the same congeners. The non-persistent and the estrogenic PCB variables were less strongly correlated with other PCB variables. Correlations of PCBs with *p,p'*-DDE were far lower. Of the PCB groups only the persistent group was correlated with HCB and the persistent group also was the PCB group most highly correlated with *p,p'*-DDE. Lead was not correlated with any of the PCB variables or with *p,p'*-DDE or HCB (results not shown).

References

DeCaprio AP, Tarbell AM, Bott A, Wagemaker DL, Williams RL, O'Hehir CM. 2000. Routine analysis of 101 polychlorinated biphenyl congeners in human serum by parallel dual-column gas chromatography with electron capture detection. J Anal Toxicol 24:403-420.

DeCaprio AP, Johnson GW, Tarbell AM, Carpenter DO, Chiarenzelli JR, Morse GS, et al. 2005. Polychlorinated biphenyl (PCB) exposure assessment by multivariate statistical analysis of serum congener profiles in an adult Native American population. Environ Res 98:284-302.

Supplemental Material, Table S1. Correlations of toxicants.

	**Σ16PCBs**	**Σ8PerPCBs**	**Σ6NonPerPCBs**	**Σ7 Estrogenic PCBs**	**Anti-estrogenic PCB**	**Σ4MOPCBs**	**Σ8DOPCBs**	**Σ3TTOPCBs**	***p,p'*-DDE**	**HCB**	**Pb (μg/dL)**
Σ16PCBs	-	0.94**	0.81**	0.97**	0.53**	0.88**	0.98**	0.88**	0.43**	0.14	0.05
Σ8PerPCBs	0.94**	-	0.57**	0.87**	0.48**	0.83**	0.94**	0.77**	0.56**	0.20*	0.03
Σ6NonPerPCBs	0.81**	0.57**	-	0.86**	0.48**	0.70**	0.78**	0.82**	0.07	0.02	0.10
Σ7 Estrogenic PCBs	0.97**	0.87**	0.86**	-	0.46**	0.81**	0.97**	0.86**	0.34**	0.12	0.07
Anti-estrogenic PCB	0.53**	0.48**	0.48**	0.46**	-	0.67**	0.44**	0.51**	0.06	-0.05	0.16
Σ4MOPCBs	0.88**	0.83**	0.70**	0.81**	0.67**	-	0.79**	0.81**	0.40**	0.06	0.03
Σ8DOPCBs	0.98**	0.94**	0.78**	0.97**	0.44**	0.79**	-	0.84**	0.44**	0.17	0.04
Σ3TTOPCBs	0.88**	0.77**	0.82**	0.86**	0.51**	0.81**	0.84**	-	0.29**	0.09	0.11
p,p'-DDE	0.43**	0.56**	0.07	0.34**	0.06	0.40**	0.44**	0.29**	-	0.41**	-0.05
HCB	0.14	0.20*	0.02	0.12	-0.05	0.06	0.17	0.09	0.41**	-	0.13
Pb (μg/dL)	0.05	0.03	0.10	0.07	0.16	0.03	0.04	0.11	-0.05	0.13	-

All organochlorines are measured in ppb. $^{**}p \leq 0.0001$; $^{*}p \leq 0.05$.

Supplemental Material, Table S2. Predictors of testosterone levels in adolescent males: Results of the multivariate regression analysis (n = 120; R^2 = 71%).

**Covariate**	**β (95% CIs)**	***p***
Tanner Stages I & II vs. III	-1.84 (-2.41, -1.27)	< 0.001
Tanner Stages IV & V vs. III	1.19 (0.60, 1.79)	< 0.001
Height-for-age *z*-score	-0.08 (-0.27, 0.12)	0.44
Weight-for-age *z*-score	0.06 (-0.12, 0.24)	0.53
Breastfeeding (y/n)	0.01 (-0.41, 0.44)	0.95
Childs Alcohol Use (y/n)	0.58 (-0.29, 1.45)	0.19
Childs Cigarette Use (y/n)	0.53 (-0.22, 1.27)	0.17
Cholesterol (mg/dL)	-0.01 (-0.01, 0.00)	0.18
Triglycerides (mg/dL)	< 0.01 (-0.01, 0.01)	0.92
Pb (µg/dL)	-0.05 (-0.22, 0.11)	0.55
p,p'-DDE (ppb)	0.52 (-0.05, 1.09)	0.07
HCB (ppb)	0.05 (-0.48, 0.58)	0.85
Σ16PCBs (ppb)[b]	-0.56 (-1.08, -0.05)	0.03

[a]Testosterone, cholesterol, triglycerides, lead, p,p'-DDE, HCB and PCB variables are ln-transformed. [b]Congeners with ≥ 50% detection rate; Sum of IUPAC#s 52, 70, 74, 84, 87, 95, 99, 101[+90], 105, 110, 118, 138[+163+164], 149[+123], 153, 180, 187. Bracket indicates 'minor' coeluting congener based on Aroclor concentration (Hansen, 1999). Values < MDL were imputed from the estimated distribution < MDL.
